# Global research priorities for urban health

**DOI:** 10.2471/BLT.22.289135

**Published:** 2022-12-01

**Authors:** Nathalie Roebbel, Thiago Herick de Sa, Maria Neira, Etienne Krug

**Affiliations:** a Department of Social Determinants of Health, World Health Organization, Avenue Appia 20, 1211 Geneva 27, Switzerland.; b Department for Environment, Climate Change and Health, World Health Organization, Geneva, Switzerland.

By 2050, more than two thirds of the world’s population will live in urban areas,^1^ presenting governments and city authorities with the daunting challenge of ensuring that urban dwellers have equitable access to safe and sustainable transport systems, and healthy, sustainable living and working environments. To meet this challenge, Member States and nongovernmental organizations have called on the World Health Organization (WHO) to support implementation of multisectoral interventions to improve the health of urban residents. In response, WHO has renewed and enhanced its organizational focus on this area^2^ and developed the Urban Health Research Agenda, a set of global research priorities for 2022–2032.^3^ The agenda includes many of WHO’s focus areas, including environmental health, tobacco, housing, healthy diets, physical activity, healthy ageing, and emergency preparedness and response, with the overarching goal of supporting governments, actors and communities in achieving their health, equity and sustainability targets. This agenda is expected to have a medium- to long-term impact at global and subnational levels by identifying gaps in urban health research; developing global research priorities that address these gaps and strengthening research methods; and providing evidence for the development of multisectoral interventions that promote urban health.^4^

The Urban Health Research Agenda has four priorities. The first priority is to strengthen links between the research findings and actions to promote urban health. Doing so involves mapping the evidence base on existing urban health interventions and their enabling factors and health impacts, and appraising other factors. These factors include existing urban health resource mobilization strategies; evidence on norms and regulations; evidence for safeguarding health through its economic and commercial determinants; and effective models for citizen participation in urban health decision-making. This priority also involves exploring the application of innovative technologies for measuring urban health risks and improving outcomes, methods for more effective research and knowledge translation and approaches for health integration into broader urban agendas.

The second priority is to build city-level evidence on the relationship between policy, environmental, economic and social factors in urban environments and health outcomes. This priority involves investigating local governance approaches to healthy urban development and policy-making, identifying enabling factors and impacts on health, and considering the relationships between neighbourhood factors and social determinants of health. This priority also involves appraising evidence on access to health services and methods for engaging local health systems, and research on the impact of geographical disparities within cities on residents in vulnerable conditions. This priority calls for exploring how population and stakeholder groups subjectively perceive urban health risks in specific city contexts and for gaining a clearer picture of the association between urban exposures and health across the life course.

The third priority is to generate evidence on under-researched thematic areas. This priority aims to strengthen evidence on the relations between climate change and urban health; on effective strategies for preparing, responding and adapting to emergencies in cities; on strategies to combat health disinformation and misinformation; and on the drivers of and interventions to address urban mental health outcomes and interventions to prevent accidents and injuries in cities. This priority also calls for stronger connections between existing global research frameworks, such as Planetary Health^5^ and One Health, that address biodiversity and the links between natural environments and health.

The fourth priority is to generate evidence on under-researched population subgroups. This priority involves exploring how urban inequities compound neighbourhood risks and outcomes, and how addressing them can improve health equity and outcomes. This priority calls for data indicators for monitoring and evaluating urban health interventions directed at specific population groups and on equity outcomes. In addition, it calls for investigating the relationship between physical and social urban environmental changes, exposures, policies and outcomes for populations in vulnerable conditions such as migrants and internally displaced groups, women and girls, older persons and people living with disabilities. This priority also provides for investigating the relationship between land use and zoning regulations and local health inequities.

As the research agenda is implemented, WHO and its partners will propose additional processes for the revision and possible expansion of the research questions. The WHO Urban Health Research Agenda implementation will engage actors outside the health sector such as those dealing with urban planning, transport and housing, and explore synergies with existing commitments, particularly the sustainable development goals. Future activities will emphasize the engagement and capacity-building of key stakeholders from governments, funding bodies, civil society and academia, to ensure that health research topics and questions are framed with a greater emphasis on the needs of urban dwellers.
